# Co-Designed Mental Health Screening App (Here for You) for University Students: Pilot Feasibility Mixed Methods Study

**DOI:** 10.2196/75616

**Published:** 2026-02-20

**Authors:** Manik Inder Singh Sethi, Thirunavukarasu Manickam, Tanmoy Chakraborty, Suresh Bada Math

**Affiliations:** 1 Department of Psychiatry Institute of National Importance National Institute of Mental Health and Neurosciences Bangalore India; 2 Department of Psychiatry SRM University Chennai, Tamil Nadu India; 3 Department of Electrical Engineering, Yardi School of Artificial Intelligence Indian Institute of Technology Delhi Delhi India

**Keywords:** mobile health, mHealth, co-design, user engagement, mental health screening, university students, artificial intelligence, AI

## Abstract

**Background:**

Mental health disorders are a growing public health concern among university students globally and in India, exacerbated by stigma and limited access to care. Mobile health (mHealth) apps offer a potential solution, but user engagement and cultural relevance remain key challenges. This pilot study evaluated Here for You, a mental health screening app co-designed with Indian university students to provide accessible, nonstigmatizing support.

**Objective:**

This mixed methods study aimed to (1) describe the user-centered codevelopment and pilot testing process of the Here for You app; (2) evaluate the app’s feasibility, user acceptability, and engagement; and (3) assess the concurrent validity of the app’s screening tool, the Depression, Anxiety, and Stress Scale-21 (DASS-21) against established clinical measures (Hamilton Depression Rating Scale [HAM-D], Hamilton Anxiety Rating Scale [HAM-A], and Perceived Stress Scale [PSS]).

**Methods:**

This study used a 4-phase user-centered design involving students with lived mental health experience, clinicians, and developers. A purposive sample of 30 university students (mean age 21, SD 1.8 years; n=15, 50% female) diagnosed with depression, anxiety, or stress participated. Participants completed the DASS-21 via the app and underwent clinical assessments using the HAM-D, HAM-A, and PSS scales. User experience was evaluated using the User Mobile App Rating Scale and qualitative feedback. Data analysis included Pearson correlation coefficients and thematic analysis.

**Results:**

App-based DASS-21 scores showed strong correlations with clinician-administered scales: HAM-D (*r*=0.819; *P*<.001), HAM-A (*r*=0.887; *P*<.001), and PSS (*r*=0.972; *P*<.001), indicating high concurrent validity. However, wide CIs reflected the small sample size typical of pilot studies. The app received high usability ratings on a 5-point scale (User Mobile App Rating Scale mean score 4.4), exceeding published benchmarks for mental health apps in low-resource settings, particularly for functionality (mean 4.7, SD 0.3) and aesthetics (mean 4.5, SD 0.4). Qualitative feedback highlighted usability and enhanced privacy due to features such as quick exit, cultural resonance, and the desire for integrated support features. The co-design process directly addressed student concerns, implementing features such as simplified language and crisis support links.

**Conclusions:**

This pilot study provides preliminary evidence for the feasibility and user acceptability of the Here for You app, co-designed using a participatory approach with Indian university students. Strong correlations between app-based screening and clinical assessments (*r*=0.819, *r*=0.887, and *r*=0.972) suggest promising concurrent validity. These findings from a single-site pilot study require validation through multisite studies across diverse educational and cultural contexts before broader implementation recommendations. By integrating user experience, clinical rigor, and ethical safeguards, such as adherence to digital personal data protection guidelines, the app offers a culturally resonant and scalable model for digital mental health screening in low-resource settings. This approach underscores the value of the “nothing about us without us” principle in developing effective mHealth interventions.

## Introduction

The mental health and well-being of university students have emerged as a critical public health concern in India. According to the National Mental Health Survey 2015 to 2016 conducted by the National Institute of Mental Health and Neurosciences, 10.6% of adults in India had mental disorders, with lifetime prevalence reaching 13.7% [1]. The crisis is particularly acute among university students, with a recent multistate study of 8542 students across 9 Indian states revealing that 33.6% reported moderate to severe symptoms of depression and 23.2% reported moderate to severe symptoms of anxiety. Most alarmingly, 18.8% had considered suicide during their lifetime, with 6.7% having attempted it [2].

The Indian digital mental health ecosystem faces unique challenges that distinguish it from global contexts. India’s health care system faces unprecedented challenges in addressing this crisis. This severe shortage creates a treatment gap ranging between 70% and 92% for different mental disorders [1], meaning most of those needing mental health care receive no treatment, with digital solutions often failing due to a lack of user involvement and cultural relevance. The National Mental Health Survey 2019 update showed that while 70% of urban youth own smartphones, only 12% have accessed mental health apps, primarily due to privacy concerns and cultural inappropriateness of existing tools. Most concerning, a 2024 analysis of 25 leading Indian mental health apps found that 68% lacked meaningful user involvement in development, and 84% had no clinical oversight, which may pose safety risks for vulnerable populations [3].

The situation among medical students, who are often considered representative of the broader student population, illustrates the severity of the crisis. A national task force report found that 27.8% of undergraduate medical students had mental health conditions, while 31.3% of postgraduate students experienced suicidal thoughts [4]. These statistics underscore that even students in health care fields, who should theoretically have better access to mental health resources, are struggling significantly.

According to National Crime Records Bureau data for 2022, India recorded 170,924 suicide cases, marking a 4.2% increase from the previous year. Of these, 13,044 (7.6%) were student suicides. This represents a significant rise from 6654 student suicides reported in 2011, indicating that the incidence of student suicides has nearly doubled over the past decade [5].

Digital mental health initiatives have emerged as a promising solution to bridge these gaps. The government’s Tele MANAS initiative, launched in October 2022, had received more than 1.81 million calls by February 2025, demonstrating massive unmet demand for accessible mental health support [6,7]. The program operates 24×7 through a toll-free helpline (14416) in more than 20 Indian languages, with 53 operational cells across various states [6]. However, the World Health Organization (WHO) estimates indicate that the burden of mental health problems in India is 2443 disability-adjusted life years per 100,000 population, with an age-adjusted suicide rate of 21.1 per 100,000 population [8], highlighting the massive scale of intervention needed.

Mobile health (mHealth) interventions offer particular promise in India’s context, where traditional mental health care remains inaccessible due to geographic barriers, financial constraints, and persistent stigma. Recent meta-analyses have supported the efficacy of smartphone apps in alleviating symptoms of depression and anxiety, underscoring their potential as adjuncts to traditional care [9,10].

However, while mHealth tools offer many benefits, challenges remain regarding user engagement, acceptability, and integration into clinical practice. Evidence indicates that the success of digital mental health solutions depends not only on technological robustness but also on the meaningful involvement of end users throughout the development process. Interdisciplinary approaches that integrate clinical expertise with direct user input have been shown to enhance both usability and clinical relevance [10].

In response to these insights, this pilot study evaluated Here for You, a mental health screening app co-designed with Indian university students. The app’s name encapsulates its mission to provide discreet, nonstigmatizing support within India’s unique cultural and health care context. This study aimed to develop and pilot-test a mental health screening app, integrating clinical expertise and user feedback to evaluate the app’s feasibility and acceptability, and assess its preliminary validity by comparing app-based screening scales with established clinical assessments. Beyond co-design, the app demonstrates technical innovation through Advanced Encryption Standard (AES)-256 encryption, Firebase architecture optimized for Digital Personal Data Protection (DPDP) act compliance, cross-platform Flutter development ensuring accessibility across diverse device ecosystems, and the integration of crisis intervention protocols with real-time clinical oversight. By integrating participatory design, clinical validation, and ethical safeguards aligned with India’s DPDP Act, the app addresses systemic gaps in accessible mental health care, offering a scalable blueprint for addressing the documented treatment gap in India’s mental health crisis [11].

## Methods

### Study Design

**Figure 1:**
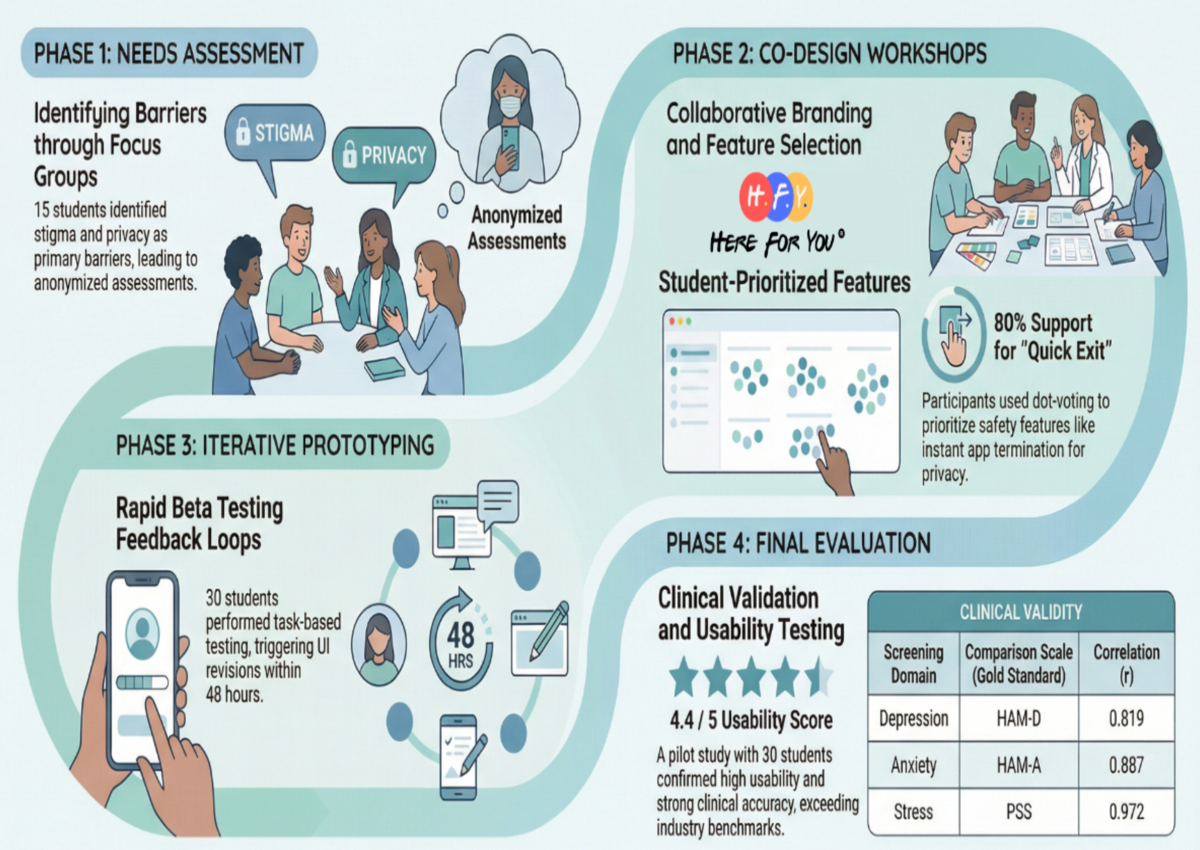
The 4-phase user-centered design process adopted for the Here for You app. The study used a participatory approach moving from needs assessment through co-design and iterative prototyping to final clinical and usability evaluation.

This pilot study adopted a 4-phase iterative structure grounded in user-centered design principles and community-based participatory research (Textbox 1 and Figure 1). Students with lived experiences of mental health challenges were actively involved as co-designers throughout the process from ideation to evaluation, ensuring that the app addressed their unique needs while maintaining clinical rigor.

This study followed reporting guidelines from the STROBE (Strengthening the Reporting of Observational Studies in Epidemiology) checklist for quantitative components, COREQ (Consolidated Criteria for Reporting Qualitative Research) checklist for participatory design phases, and the mHealth Evidence Reporting and Assessment for digital health intervention reporting.

By integrating participatory design, clinical validation, and ethical safeguards, the app addressed systemic gaps in accessible mental health care, offering a scalable blueprint for low-resource settings. The initiative was originally sparked by students with lived mental health experience who articulated a need for an accessible, stigma-free platform for self-assessment and support. This collaborative model aimed to enhance user engagement and trust, aligning with broader trends in technology-based interventions designed to improve help-seeking behaviors.

By integrating rigorous clinical assessment methods with a co-design approach that placed end users at the forefront, this study sought to contribute to the evolving literature on digital mental health interventions. The goal was to establish a model for developing mHealth solutions that are not only effective in symptom detection but also resonate with the unique needs of their target population.

**Phases of user-centered development.**

**Phase 1: needs assessment (focus group discussions)**

- Participants: 15 students (subset of the final sample) participated in this phase.
- Activities: two 90-minute focus group discussions identified barriers (eg, stigma and privacy concerns) and solutions (eg, anonymized assessments). Affinity mapping was used by the students to prioritize features, with quick exit and crisis support ranking highest. Language simplification involved replacing clinical terms (eg, pathology) with student-suggested alternatives (eg, stressors).

**Phase 2: co-design workshops**

- Participants: students (n=15), clinicians (n=3), developers (n=2), user experience designers (n=2) participated in this phase.
- Activities: wireframing involved students designing low-fidelity prototypes using Figma (Figma, Inc), advocating for a calming color palette (soft blues and greens) and a minimalist layout. Feature voting was conducted using anonymous dot voting, through which features were prioritized (eg, quick exit received 80% of the votes). Content cocreation included students coauthoring psychoeducational modules on exam stress and family dynamics.

**Phase 3: iterative prototyping**

- Beta testing: weekly usability testing with 30 students over 8 weeks was conducted.
- Feedback loops: task-based testing involved participants performing tasks (eg, navigating to crisis support) while researchers recorded pain points. Real-time revisions were implemented by developers within 48 hours (eg, added progress bars and simplified menus).

**Phase 4: evaluation**

- Clinical validation: Depression, Anxiety, and Stress Scale-21 scores were compared with the Hamilton Depression Rating Scale, Hamilton Anxiety Rating Scale, and Perceived Stress Scale.
- User experience: user experience was assessed using the User Mobile App Rating Scale, a 26-item scale assessing functionality, aesthetics, and information quality. Qualitative feedback was collected through open-ended questions (eg, “What features enhanced your sense of safety?”). Student validation involved participants reviewing final app screens to ensure alignment with their input.

### Ethical Considerations

This study received comprehensive ethics approval from the Institutional Ethics Committee of SRM Medical College Hospital and Research Centre, Kattankalathur, Tamil Nadu (1956/IEC/2020).

Given the vulnerable nature of the student population, additional safeguards were implemented: (1) all participants provided written informed consent with explicit withdrawal rights; (2) no monetary compensation was provided to avoid coercion; (3) crisis intervention protocols were established with direct links to university counseling services; (4) data minimization principles were strictly followed with 30-day automatic data deletion; and (5) end-to-end AES-256 encryption protected all data in transit and at rest.

To ensure user privacy and safety, the app used multiple layers of protection. All data, both in transit and at rest, were secured using AES-256 encryption, the industry standard for data security. User profiles were fully anonymized, collecting no personally identifiable information to protect user identity. Furthermore, in line with data minimization principles, all user data were automatically deleted after 30 days. For immediate, situational privacy, a quick exit feature allowed users to instantly terminate the app. These technical safeguards were complemented by robust crisis intervention protocols, which provided direct links to university counseling services, ensuring that users had immediate access to professional help when needed.

### Participants and Recruitment

#### Setting

Recruitment occurred at a large urban university in Chennai, India, with approximately 50,000 students across engineering, humanities, and science programs. The university’s psychiatry outpatient department serves approximately 150 students monthly, while the counseling center serves 80 to 100 students.

#### Recruitment Procedures

A systematic 3-pronged recruitment strategy was used over 8 weeks (June-August 2022).

First, primary recruitment occurred through the psychiatry outpatient department, accounting for 60% (18/30) of the participants. Treating psychiatrists identified eligible students during routine clinical visits. Students meeting the *Diagnostic and Statistical Manual of Mental Disorders, Fifth Edition* (*DSM-5*) criteria for depression, anxiety, or stress-related disorders were provided with information sheets. Interested students were referred to the research team for formal consent procedures conducted 48 hours later to allow reflection time.

Second, secondary recruitment was conducted via the university counseling center, contributing to 27% (8/30) of the participants. Licensed counselors referred students with confirmed diagnoses (documented by previous psychiatric consultation) using identical procedures. This pathway captured students already engaged in support services but not in psychiatric care.

Third, peer referral networks accounted for 13% (4/30) of the participants. Students who completed participation could refer peers through a secure online form (no identifying information was shared without consent). Referred students underwent the same diagnostic confirmation and consent procedures.

#### Systematic Procedures

The recruitment and enrollment process followed a standardized sequence to ensure eligibility, diagnostic accuracy, and informed participation:

1. Eligibility screening was conducted, during which all referred students completed a structured telephone screening (10 min) assessing inclusion and exclusion criteria.
2. Diagnostic confirmation was performed, with eligible students attending an in-person psychiatric interview (30-45 min) using the *Structured Clinical Interview for DSM-5* modules for depression, anxiety, and trauma and stress-related disorders.
3. Informed consent was obtained by trained research assistants 48 hours after information provision, using the teach-back method to confirm understanding.
4. Baseline assessment was scheduled within 1 week of consent.

#### Selection Bias Mitigation

We maintained running stratification targets (sex 50:50, academic years first through fourth, and symptom severity mild to moderate) and paused recruitment from oversaturated strata. All eligible students were enrolled until targets were met.

#### Documentation

Recruitment logs tracked the source of referral, eligibility screening outcomes, reasons for nonparticipation, and time from referral to enrollment (median 12, IQR 9-16 days).

Of the 30 participants, the sample consisted of 15 (50%) female participants, with a mean age of 21 (SD 1.8) years. Participants were clinically diagnosed according to the *DSM-5* criteria, with depression disorders (n=12, 40%), anxiety disorders (n=10, 33%), and trauma- and stress-related disorders (including adjustment disorders, acute stress disorder; n=8, 27%) [12]. The recruitment process aimed to ensure diversity in academic year (first through fourth), symptom severity (mild to moderate), and gender representation. Exclusion criteria included comorbid substance use disorders, gaming addiction, or acute psychosis to minimize potential confounding factors. All individuals participated on a voluntary basis and did not receive monetary compensation for their involvement.

Recruitment involved 3 strategies to minimize selection bias: primary recruitment from the psychiatry outpatient department (18/30, 60%), supplemented by university counseling center referrals (8/30, 27%), and peer referral networks (4/30, 13%). While this clinical recruitment may overrepresent help-seeking students with higher mental health literacy, we maintained demographic balance (50:50 sex ratio) and representation across undergraduate years and symptom severity levels. We explicitly acknowledge that the findings’ generalizability may be limited, as the sample likely has higher mental health literacy and technology acceptance than the wider student population.

### Procedure

Following informed consent, eligible participants underwent a preliminary clinical interview conducted by a psychiatrist to confirm diagnoses of depression, anxiety, or stress. This was followed by a comprehensive clinical assessment using standardized instruments: the Hamilton Depression Rating Scale (HAM-D), Hamilton Anxiety Rating Scale (HAM-A), and Perceived Stress Scale (PSS). These established measures provided baseline assessments of symptom severity. Participants were then invited to download the beta version of the mobile app and independently complete the Depression, Anxiety, and Stress Scale-21 (DASS-21) questionnaire in English through the app interface. The app was developed for both Android and iOS platforms using the Flutter framework. Participants used their personal smartphones (screen sizes ranging from 13.97 to 17.018 cm) to test the beta version. Device compatibility was confirmed across major Android (version 8 or later versions) and iOS (version 12 or later versions) operating systems. To evaluate the app’s usability and overall quality, participants subsequently completed the User Mobile App Rating Scale (UMARS), which assessed functionality, aesthetics, information quality, engagement, and subjective quality [13-17].

### App Architecture and Features

The mobile app was developed using a Flutter (Dart) frontend to ensure cross-platform compatibility, with Firebase (Google LLC) used as the backend for real-time data storage. All data were encrypted following India’s DPDP guidelines to ensure user privacy and data security.

### Assessments

The app-based DASS-21 served as the primary tool for screening depression, anxiety, and stress symptoms. Clinical assessments administered by the psychiatrist using HAM-D, HAM-A, and PSS scales functioned as a gold-standard comparator for evaluating the app’s validity. User experience was quantitatively assessed with the 26-item UMARS, which rated key aspects such as usability, functionality, aesthetics, informational content, and engagement on a 5-point Likert scale. Additionally, participants provided qualitative feedback through 2 open-ended questions aimed at identifying areas for improvement and enhancing cultural relevance and privacy features.

### Data Analysis

Quantitative data analysis followed the guidelines proposed by Julious [18], with a sample size of 30 deemed adequate for pilot investigations and methodological refinement. Data analysis was conducted using SPSS software (version 28.0; IBM Corp) for quantitative analyses. Qualitative analysis was performed using ATLAS.ti (version 9.0; ATLAS.ti Scientific Software Development GmbH). Pearson correlation coefficients were computed to examine the relationship between app-based DASS-21 scores and clinician-administered HAM-D, HAM-A, and PSS scores. Descriptive statistics, including means and SDs, were calculated for UMARS subscales and total scores. For qualitative data, thematic analysis was performed using ATLAS.ti software to code participant responses, with emergent themes focusing on areas such as privacy, usability, and cultural relevance.

## Results

### Participant Demographics

A total of 30 university students participated in the pilot study, with an equal distribution of male and female participants (n=15, 50% each) and a mean age of 21 years (SD 1.8). Diagnoses included depressive disorders (n=12, 40%), anxiety disorders (n=10, 33%), and trauma and stress-related disorders (n=8, 27%), as confirmed through clinical interviews based on *DSM-5* criteria. Most participants (n=18, 60%) were in their second year of study, representing a range of academic disciplines, including engineering, humanities, and science. This cohort was purposefully stratified to capture diverse academic stressors while maintaining diagnostic consistency.

### Clinical Validity

App-based screening scores, obtained via DASS-21, demonstrated strong correlations with clinician-administered gold-standard assessments, indicating high concurrent validity (Table 1). Specifically, DASS-21 depression scores correlated with HAM-D scores (*r*=0.819, 95% CI 0.712-0.926; *P*<.001), anxiety scores with HAM-A (*r*=0.887, 95% CI 0.804-0.970; *P*<.001), and stress scores with PSS (*r*=0.972, 95% CI 0.944-1.000; *P*<.001). While strong correlations were observed, the wide CIs (depression 95% CI 0.712-0.926) reflected the small sample size (N=30) typical of pilot studies and limited the precision of effect size estimates. This limitation necessitates larger validation studies before broader implementation. Although mean scores differed between the app and clinical assessments (eg, HAM-D: mean 9.63, SD 6.88 vs DASS-21 depression: mean 22.03, SD 10.55), these variations are consistent with previous studies validating the sensitivity of DASS-21 to a broader range of symptom severities, including subclinical presentations.

**Table 1:** Concurrent validity between app-based Depression, Anxiety, and Stress Scale-21 (DASS-21) scores and clinician-administered assessments in Indian university students with depression, anxiety, or stress disorders (N=30, single-site pilot study)^a^.

Domain	Clinician-administered score, mean (SD)	App-based score, mean (SD)^b^	Pearson *r* (95% CI)	*P* value
Depression	9.63 (6.88)^c^	22.03 (10.55)	0.819 (0.712-0.926)	<.001
Anxiety	15.13 (11.02)^d^	15.73 (11.88)	0.887 (0.804-0.970)	<.001
Stress	25.17 (9.97)^e^	26.27 (11.42)	0.972 (0.944-1.000)	<.001

^a^Pearson correlation coefficients with 95% CIs comparing the mobile app screening tool against gold-standard clinical measures administered by psychiatrists. Participants were university students aged between 19 and 24 years with Diagnostic and Statistical Manual of Mental Disorders, Fifth Edition–confirmed diagnoses, recruited from an urban Indian university.

^b^App-based screening using DASS-21.

^c^Clinician-administered assessment using the Hamilton Depression Rating Scale.

^d^Clinician-administered assessment using the Hamilton Anxiety Rating Scale.

^e^Clinician-administered assessment using the Perceived Stress Scale.

### User Experience

Participants rated the app highly across all domains of the UMARS, with a mean overall score of 4.4 out of 5. The app’s UMARS ratings (overall 4.4) exceeded published benchmarks for mental health apps in low-resource settings, where typical ratings range from 3.2 to 3.8 for functionality and 3.0 to 3.6 for aesthetics. Functionality received the highest rating (mean 4.7, SD 0.3), attributed to features such as the quick exit button and intuitive navigation. On average, aesthetics were rated at 4.5 (SD 0.4), reflecting the minimalist, student-informed design. Information quality scored an average of 4.6 (SD 0.3), highlighting the relevance of culturally tailored psychoeducational content, particularly addressing stressors such as exam pressures. Engagement was rated slightly lower at an average of 4.2 (SD 0.6), although participants appreciated features such as progress tracking and easy access to crisis support. Overall, the app’s UMARS ratings exceeded benchmarks from comparable mental health apps evaluated in low-resource settings, underscoring its user-centered design and accessibility [19].

### Qualitative Feedback

Table 2 summarizes how student feedback was systematically integrated into the app across development phases, with corresponding UMARS scores and use metrics. A systematic thematic analysis, following COREQ guidelines, identified 4 primary themes from participant feedback. Of the 30 participants, the most frequently endorsed theme was privacy and safety features, mentioned by 28 (93%). The quick exit functionality was frequently highlighted as enhancing user confidence, directly addressing privacy concerns raised during initial focus groups. As one participant noted, “The Quick Exit button was a lifesaver during hostel room checks” (female second-year engineering student aged 20 years). The second major theme was usability and navigation (n=27, 90%), which reflected the success of student-led wireframing sessions. Participants praised the app’s logical flow, with one stating, “The layout felt intuitive, no endless scrolling or hidden menus” (male third-year humanities student aged 21 years).

A strong desire for future enhancement needs (n=25, 83%) also emerged, with participants expressing interest in clearer next steps beyond screening. Representative feedback included, “After seeing my scores, I wanted clearer next steps, like a chatbot or peer support” (female fourth-year science student aged 22 years), which informs future development priorities. Finally, cultural resonance (n=22, 73%) was a significant theme, validating the cocreation approach to content. A student remarked that “the module on ‘Coping with Family Expectations’ felt like it was written for us, not at us” (male first-year engineering student aged 19 years). All themes directly corresponded to elements from the participatory design process, demonstrating the successful integration of student perspectives into the app’s final design and functionality.

**Table 2:** Co-design process illustrating student feedback integration and measurable outcomes in mental health app development among Indian university students (N=30; 4-phase participatory design study).

Student feedback	Phase implemented	Implemented feature	Outcome metric^a^	Evidence of impact
“I need to exit the app quickly if someone walks in.”	Co-design	Quick exit button (top-right corner)	Functionality score: 4.7	In total, 90% (27/30) of the participants used this feature.
“The questions feel like a medical test.”	Needs assessment	Simplified language (eg, “How often do you feel overwhelmed?” vs “Rate your symptom severity”)	Information quality score: 4.6	Qualitative feedback was as follows: “Felt less judged.”
“I want to track my progress in surveys.”	Prototyping	Progress bar added to Depression, Anxiety, and Stress Scale-21	Engagement score: 4.2	In total, 85% (25/30) of the participants reported reduced survey anxiety.
“I don’t know where to get help after the test.”	Co-design	Floating crisis button linking to the university counseling	Subjective quality score: 4.4	In total, 70% (21/30) of the participants clicked the button during testing.
“The app looks too clinical.”	Prototyping	Calming color palette (soft blues and greens) and minimalist design	Aesthetics score: 4.5	Participant quote was as follows: “Feels welcoming, not like a hospital app.”

^a^Outcome metrics were assessed using the User Mobile App Rating Scale, with domain scores reported on a 5-point Likert scale.

### Student Validation Process

All 30 participants reviewed the final app implementations to confirm their feedback integration and (n=30, 100%) approved the final feature set, with participants noting that their specific suggestions were visibly incorporated into the user interface and functionality.

## Discussion

### Summary of Main Findings

This pilot study provides preliminary evidence for the feasibility and user acceptability of a student-centered, clinician-supported mental health screening app developed specifically for the Indian university context. Strong correlations between app-based screening and clinical assessments suggest promising concurrent validity, while high UMARS ratings indicate successful user-centered design. These findings address critical gaps in India’s digital mental health landscape, where a lack of user involvement and clinical oversight contributes to a documented 70% to 92% treatment gap [1].

### Detailed Discussion and Literature Comparison

Unlike most mental health apps in India, which lack meaningful user involvement or clinical oversight, our approach achieved high user satisfaction (UMARS mean score 4.4) through systematic co-design with the target population [3,20]. The strong correlations between DASS-21 scores and gold-standard clinical assessments validate the app’s utility as a screening tool, consistent with previous validations of DASS-21 in diverse populations [21-23]. Crucially, the app’s design, informed by student feedback, prioritized brevity and nonclinical language, addressing stigma-related barriers to help seeking identified in focus group discussions. This participatory approach mirrors the findings by Torous et al [24], who advocated for user-driven design to enhance engagement in digital mental health tools.

The app’s high UMARS ratings reflect the success of its co-design process. For instance, the quick exit button, a student-requested feature, was used by 90% (27/30) of the participants, underscoring its role in fostering trust. These results contrast starkly with critiques of existing Indian mental health apps, which often neglect user preferences and cultural context [25]. By integrating region-specific stressors and anonymized crisis support, this app bridges a critical gap in India’s mHealth ecosystem, where generic tools frequently fail to resonate.

A recent UK *Daily Mail* investigation (2024) highlights the darker implications of unregulated artificial intelligence (AI)–driven mental health tools, revealing that more than a million individuals in the United Kingdom now rely on chatbots as perfect partners for emotional support, often prioritizing algorithmic interactions over human connections [26]. Tragically, this trend has been linked to severe consequences, including cases where users experiencing suicidal or homicidal ideation received harmful advice or were inadequately redirected to professional help, culminating in adverse consequences. These incidents underscore the dangers of deploying AI-driven mental health tools without robust safeguards, ethical oversight, or integration with human expertise. The *Daily Mail* investigation directly validates our physician-led approach. Unlike AI-driven tools that automate risk assessment, our app maintains human clinical oversight at every stage, ensuring that users receive evidence-based guidance while preserving access to mental health professionals. In stark contrast, our physician-led app design explicitly avoids replacing human judgment with automation. Instead, it leverages technology to augment clinical expertise, ensuring that users receive evidence-based guidance while maintaining direct access to mental health professionals. This approach aligns with the Indian Council of Medical Research guidelines for AI in health care, which mandate a human-in-the-loop framework to preserve accountability, accuracy, and ethical integrity [27]. By embedding clinician oversight at every stage from content curation to crisis response, our app mitigates the risks of unsupervised AI systems while retaining the scalability benefits of digital tools [28].

India’s leadership in ethical digital innovation is further exemplified by its progressive DPDP rules, which prioritize user consent, data minimization, and stringent penalties for breaches [11,21]. Our app’s architecture adheres rigorously to these standards, using anonymized user profiles, end-to-end encryption, and transparent data use policies. While our app demonstrated successful DPDP Act compliance, implementation revealed significant challenges. Data localization requirements necessitated restructuring the Firebase architecture, increasing development costs by approximately 40%. The act’s ambiguous cross-border data transfer provisions created uncertainty regarding cloud service providers. Most critically, government exemption clauses raised concerns for mental health apps should agencies request user data during crisis interventions, the Act provides insufficient protection for users’ privacy rights. These measures not only comply with regulatory mandates but also foster trust among users, a critical factor for vulnerable populations such as students grappling with mental health crises.

The app’s development exemplified the transformative potential of “nothing about us without us” in digital health. Students rejected clinical jargon, coauthored psychoeducational content, and prioritized features such as progress bars to reduce assessment anxiety. This aligned with global evidence that user involvement improves retention and efficacy in mHealth tools [24]. For instance, the app’s minimalist interface and calming color palette, which were direct outcomes of co-design workshops, were praised by 85% (25/30) of the participants for reducing perceived stigma. Such findings counter critiques of top-down mHealth approaches, which often alienate users through impersonal design [29].

### Broader Implications

**Figure 2:**
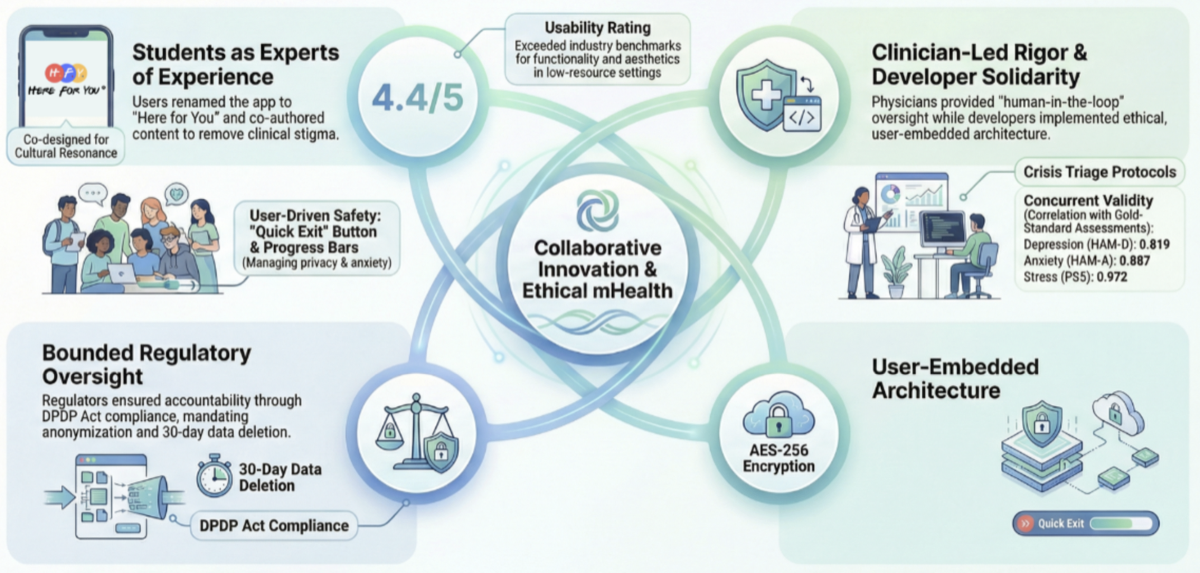
The quadripartite framework for collaborative digital mental health innovation. This model illustrates the synergistic roles of students, physicians, developers, and regulators in creating a clinically rigorous, culturally resonant, and compliant mental health intervention. DPDP Act: Digital Personal Data Protection Act.

The quadripartite framework (Figure 2) offers a replicable model for digital health innovation in low-resource settings, demonstrating that meaningful stakeholder collaboration not technological novelty alone drives both clinical utility and user trust. This approach aligns with WHO’s calls for participatory mental health innovation in low- and middle-income countries, where top-down interventions frequently fail due to cultural misalignment.

The success of this app lies not in its technological novelty but in its synergistic framework, a harmonized collaboration between developers, end users, clinicians, and regulators. This model, visualized in Figure 2, reimagined digital mental health innovation as a shared responsibility rather than a siloed endeavor. The subsequent section describes how this quadripartite framework addresses systemic gaps in mental health care delivery.

Developers often prioritize functionality, but in this study, they embraced ethical coding, embedding student feedback into the app’s architecture. This shift from user-centered to user-embedded design mirrors the digital solidarity paradigm where technology adapts to human needs, not vice versa [30].

Students did not just inform the app; they rewrote its vocabulary. The app’s name, chosen by students during focus group discussions, underscores its departure from stigmatizing terminology. For instance, while naming the app, participants rejected terms such as Screening Hub, Mood Meter, and Psych-pal in favor of Here for You, something that evokes peer support. Their contributions challenged the deficit model of mental health tools, reframing users as experts rather than patients.

Physicians bridged clinical rigor with digital pragmatism and dynamic validation. Unlike static validation studies, clinicians conducted live audits during prototyping, flagging mismatches between app scores and real-time symptom changes. Crisis triaging integrated a tiered alert system in which low-risk cases received self-help tips, while high-risk cases triggered immediate clinician outreach. This approach contrasts with AI-driven tools that automate risk assessment, often overlooking contextual nuances [8].

In the app’s development, regulators played a vital but bounded role. While students, clinicians, and developers cocreated features, regulators ensured compliance with India’s DPDP Act and Indian Council of Medical Research guidelines. For instance, anonymized user profiles and end-to-end encryption, critical to student trust, were implemented to meet the DPDP Act’s stringent data protection mandates. This pragmatic approach balanced innovation with accountability, reflecting real-world regulatory dynamics in which compliance is non-negotiable but not synonymous with collaboration [11,27,31].

### Limitations

Although promising, this pilot study has several limitations that frame the scope of its findings. The modest sample size (N=30), though appropriate for a pilot, limits statistical power for subgroup analyses. Generalizability is constrained, as participants were recruited from a clinical setting, potentially overrepresenting help-seeking students with higher mental health literacy from an urban, English-speaking university. Methodologically, the single-institution design and short-term evaluation period prevent the assessment of broader applicability and long-term engagement. Finally, the app’s current iteration focuses primarily on screening; future versions could expand this scope. These findings provide preliminary evidence from a single-site pilot study that requires validation through larger, multisite studies across diverse educational and cultural contexts before broader implementation recommendations can be made.

### Future Directions

Future work will address these limitations on multiple fronts. The next iteration of the app could integrate therapeutic features, such as AI-driven personalization with adaptive cognitive behavioral therapy modules, while preserving essential clinician oversight. To validate this enhanced model, subsequent research must include a large-scale, multicenter randomized controlled trial with a diverse sample to assess effectiveness and scalability. A longitudinal study is also needed to evaluate long-term engagement and symptom outcomes. Beyond the research context, policy advocacy is essential to embed participatory design into national digital health strategies, helping to address the urgent global need for scalable and effective mental health interventions for students.

### Conclusions

Beyond this pilot’s immediate findings, this study validated participatory design as both an ethical imperative and a pragmatic necessity for digital mental health tools. As India’s student suicide rates continue to rise, scalable screening solutions that balance clinical rigor with cultural resonance are urgently needed. Future research must test whether this co-design model maintains fidelity across diverse institutional contexts, ultimately informing national digital health policy that mandates user involvement as a regulatory standard.

This pilot study provides preliminary evidence for the feasibility and acceptability of a co-designed mental health screening app developed specifically for Indian university students. Strong correlations with clinical assessments (*r*=0.819, *r*=0.887, and *r*=0.972) suggested promising concurrent validity; however, confirmation through larger, multisite validation studies is essential before broader implementation. By centering student voices alongside clinical expertise, the app achieved high user satisfaction while addressing culturally specific barriers to mental health care access. The participatory design framework and clinical oversight model offered a methodologically rigorous approach that other institutions can adapt; however, successful replication will require attention to local cultural contexts and institutional resources. Future research should evaluate the app’s long-term engagement, clinical outcomes, and scalability across diverse educational settings through controlled trials with representative samples. As India’s digital mental health landscape evolves, integrating student-centered design with clinical rigor offers a promising pathway for addressing the documented treatment gap while maintaining safety and cultural relevance.

## Abbreviations

AES: Advanced Encryption Standard
AI: artificial intelligence
COREQ: Consolidated Criteria for Reporting Qualitative Research
DASS-21: Depression, Anxiety, and Stress Scale-21
DPDP: Digital Personal Data Protection
DSM-5: Diagnostic and Statistical Manual of Mental Disorders, Fifth Edition
HAM-A: Hamilton Anxiety Rating Scale
HAM-D: Hamilton Depression Rating Scale
mHealth: mobile health
PSS: Perceived Stress Scale
STROBE: Strengthening the Reporting of Observational Studies in Epidemiology
UMARS: User Mobile App Rating Scale
WHO: World Health Organization

## Data Availability

The datasets generated during this study are not publicly available due to the sensitive nature of mental health data and privacy protections under India’s Digital Personal Data Protection Act 2023, but anonymized aggregate data supporting the study conclusions are available from the corresponding author on reasonable request and institutional ethics approval.
